# Stratification of gastric cancer risk using a deep neural network

**DOI:** 10.1002/jgh3.12281

**Published:** 2019-12-26

**Authors:** Hiroko Nakahira, Ryu Ishihara, Kazuharu Aoyama, Mitsuhiro Kono, Hiromu Fukuda, Yusaku Shimamoto, Kentaro Nakagawa, Masayasu Ohmori, Taro Iwatsubo, Hiroyoshi Iwagami, Kenshi Matsuno, Shuntaro Inoue, Noriko Matsuura, Satoki Shichijo, Akira Maekawa, Takashi Kanesaka, Sachiko Yamamoto, Yoji Takeuchi, Koji Higashino, Noriya Uedo, Takashi Matsunaga, Tomohiro Tada

**Affiliations:** ^1^ Department of Gastrointestinal Oncology Osaka International Cancer Institute Osaka Japan; ^2^ AI Medical Service Inc Tokyo Japan; ^3^ Department of Medical Informatics Osaka International Cancer Institute Osaka Japan; ^4^ Tada Tomohiro Institute of Gastroenterology and Proctology Saitama Japan; ^5^ Department of Surgical Oncology, Graduate School of Medicine The University of Tokyo Tokyo Japan

**Keywords:** artificial intelligence, convolutional neural network, endoscopy, gastric cancer

## Abstract

**Background and Aim:**

Stratifying gastric cancer (GC) risk and endoscopy findings in high‐risk individuals may provide effective surveillance for GC. We developed a computerized image‐ analysis system for endoscopic images to stratify the risk of GC.

**Methods:**

The system was trained using images taken during endoscopic examinations with non‐magnified white‐light imaging. Patients were classified as high‐risk (patients with GC), moderate‐risk (patients with current or past *Helicobacter pylori* infection or gastric atrophy), or low‐risk (patients with no history of *H. pylori* infection or gastric atrophy). After selection, 20,960, 17,404, and 68,920 images were collected as training images for the high‐, moderate‐, and low‐risk groups, respectively.

**Results:**

Performance of the artificial intelligence (AI) system was evaluated by the prevalence of GC in each group using an independent validation dataset of patients who underwent endoscopic examination and *H. pylori* serum antibody testing. In total, 12,824 images from 454 patients were included in the analysis. The time required for diagnosing all the images was 345 seconds. The AI system diagnosed 46, 250, and 158 patients as low‐, moderate‐, and high risk, respectively. The prevalence of GC in the low‐, moderate‐, and high‐risk groups was 2.2, 8.8, and 16.4%, respectively (*P* = 0.0017). Three experienced endoscopists also successfully stratified the risk; however, interobserver agreement was not satisfactory (kappa value of 0.27, indicating fair agreement).

**Conclusion:**

The current AI system detected significant differences in the prevalence of GC among the low‐, moderate‐, and high‐risk groups, suggesting its potential for stratifying GC risk.

## Graphical abstract

The artificial intelligence system used in this study detected significant differences in the prevalence of gastric cancer (GC) among the low‐, moderate‐, and high‐risk groups, suggesting its potential for stratifying GC risk.

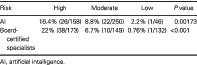

## Introduction

Gastric cancer (GC) is the fifth most common malignancy and the third leading cause of cancer‐related mortality worldwide.^1^ The numbers of new cases and fatalities are increasing because of the expansion and aging of the world population.^2^ Although advanced GC has a poor 5‐year survival rate of <25%,^3^ early detection can substantially improve both morbidity and survival. For example, nationwide mass screening programs for gastric neoplasia in Japan have reduced the related mortality.^4^ However, nationwide screening of GC is ineffective in areas with a lower incidence; this is true even in Eastern Asia, where the prevalence of *Helicobacter pylori* infection is falling.

*H. pylori* infection causes gastric mucosal atrophy and intestinal metaplasia (IM), and the risk of gastric carcinogenesis increases in line with this progression.^5^ In 2012, the first international guidelines^6^ recommended endoscopic surveillance for patients with moderate to severe atrophic gastritis (AG), marked IM in both the antrum and corpus, and dysplasia but not in patients with AG or IM limited to the antrum. However, the gold standard for defining “extensive atrophy/IM” remains unclear, and the classifications and patterns used to describe and detect these lesions have been heterogeneous and not widely reproducible.^7^, ^8^ In addition, poor interobserver variability has prevented the widespread endoscopic assessment of AG and IM.^9^

The updated Sydney System was mainly created to diagnose *H. pylori*‐associated gastritis.^10^ In addition, the Operative Link on Gastritis Assessment (OLGA)^11^, ^12^ and Operative Link on Gastric Intestinal Metaplasia Assessment (OLGIM)^13^ staging systems, based on the updated Sydney System, have recently become widely used in the West. The OLGA staging system provides a histological measure of the severity and topography of AG, whereas the OLGIM staging system is based on the severity and topography of IM. These systems have been used to identify patients at high risk of GC based on biopsy samples.^13^, ^14^, ^15^ However, biopsy confers a risk of gastrointestinal bleeding and requires a prolonged procedure time, thus increasing the burden on endoscopists and pathologists.

Accurate and objective staging of AG or IM by endoscopic observation would provide a rational measure for stratifying the GC risk. Computerized image analysis may provide a potential solution for mitigating both the variability and complexity associated with the endoscopic diagnosis of GC risk. Deep learning is a machine learning system, typically based on artificial neural networks, that aims to learn multilevel representations of data useful for making classifications. This technology has been shown to exceed human performance in visual tasks such as playing the game Go^16^ and in object recognition.^17^ More recently, it has been applied to medical fields, including the detection of gastrointestinal lesions.^18^, ^19^, ^20^, ^21^ In the current study, we developed a computerized image analysis system using deep learning to stratify the risk of GC.

## Methods

### *Preparation of training dataset*

We developed a deep learning‐based artificial intelligence (AI) system for the assessment of GC risk. The system was trained using endoscopic images taken during daily endoscopic examinations at Osaka International Cancer Institute and Tada Tomohiro Institute of Gastroenterology and Proctology (Saitama, Japan). The endoscopic procedures were mainly carried out using high‐resolution or high‐definition upper gastrointestinal endoscopes (GIF‐XP290N, GIF‐Q260J, GIF‐RQ260Z, GIF‐FQ260Z, GIF‐Q240Z, GIF‐H290Z, GIF‐H290, GIF‐HQ290, and GIF‐H260Z; Olympus, Tokyo, Japan) and video processors (CV260; Olympus), a high‐definition magnifying upper gastrointestinal endoscope (GIF‐H290Z, GIF‐H290, GIF‐HQ290, or GIF‐H260Z; Olympus) and a video processor (EVIS LUCERA CV‐260/CLV‐260 and EVIS LUCERA ELITE CV‐290/CLV‐290SL; Olympus), or a high‐resolution endoscope (EG‐L590ZW, EG‐L600ZW, or EG‐L600ZW7; Fujifilm Co., Tokyo, Japan) and a video endoscopic system (LASEREO; Fujifilm Co.). Routine inspection was mainly conducted using nonmagnified white‐light imaging.

Endoscopic images of patients who underwent endoscopic examination at Tada Tomohiro Institute of Gastroenterology and Proctology from December 2015 to April 2017 or at Osaka International Cancer Institute from April 2016 to August 2018 were used to educate the system regarding moderate‐ and low‐risk patients. The inclusion criteria were patients with a known *H. pylori* status (past infection, current infection, or no infection) or with a diagnosis of gastric atrophy by a board‐certified trainer (Noriya Uedo, Takashi Kanesaka, or Satoki Shichijo). Patients with current GC or a history of GC were excluded. Endoscopic images of patients who underwent endoscopic examination at Osaka International Cancer Institute from October 2010 to March 2016 were used to educate the system regarding high‐risk patients. The inclusion criterion was referral to our hospital for treatment of GC (Table 1). Patients with familial adenomatous polyposis, gastrostomy, or gastrectomy were excluded. Poor‐quality images resulting from less insufflation of air, bleeding, halation, blur, defocus, or mucus were also excluded from the training dataset.

**Table 1:** Clinical characteristics of the patients in the training dataset

Clinical characteristics	*n* = 7826
Age (years)	51 (16–91)
Gender, *n* (%)	
Male	3638 (47)
Female	4177 (53)
Serum antibody for *Helicobacter pylori*, *n* (%)	
Negative	5613 (72)
Positive	1592 (20)
Unknown	621 (8)
Gastric cancer, *n* (%)	
Negative	6797 (87)
Positive	1018 (13)

The groups were defined as high risk (patients with GC), moderate‐risk (patients with current or past *H. pylori* infection or gastric atrophy), and low‐risk (patients with no history of *H. pylori* infection or gastric atrophy). After selection, 20,960, 17,404, and 68,920 images were collected as the training images for the high‐, moderate‐, and low‐risk groups, respectively. All images of patients with certain risk were used as the training dataset of that risk group. For example, all images of *H. pylori*‐negative patients were used as the training dataset of the low‐risk group. These images were classified into four groups based on the location shown in the images: (i) the cardia and fornix, (ii) mainly the lesser curvature of the gastric body in the retroflex view, (iii) mainly the greater and anterior wall of the gastric body in the forward view, and (iv) the antrum. For the high‐risk group, the area of the GC or scar after endoscopic resection was marked manually by a board‐certified specialist (H.N.) at the Japan Gastroenterological Endoscopy Society using a rectangular frame to exclude it from the image data regarding GC.

### *Construction of AI system*

A deep convolutional neural network (CNN) model is a type of artificial neural network used in deep learning. The base CNN used in this study was a visual geometry group network consisting of 16 layers. The CNN learns the filters that were previously hand‐engineered in more traditional algorithms. This independence from prior knowledge and human effort represents a significant advantage of neural network models over other types of machine learning.^22^

In this study, we used the Single Shot MultiBox Detector CNN architecture without changing its algorithm. The CNN was then trained, validated, and tested using the Caffe deep learning framework, originally developed at the Berkeley Vision and Learning Center. Model training was carried out by stochastic gradient descent with a global learning rate of 0.0001, 80 epochs, and batch size of 32. Each image was resized to 300 × 300 pixels, and the bounding box was resized for optimal CNN analysis. These values were set by trial and error to ensure that all the data were compatible with the Single Shot MultiBox Detector.

### *Evaluation of AI system*

The performance of the AI system was evaluated based on the prevalence of GC in each group using an independent validation dataset of patients who underwent endoscopic examination and *H. pylori* serum antibody testing at Osaka International Cancer Institute from October 2010 to March 2016. The exclusion criteria were a history of gastrectomy, previous treatment for GC, and a previous diagnosis of GC in another hospital. For the evaluation, all images of the gastric mucosa were included in the analysis.

The trained neural network generated a diagnosis of high, moderate, or low risk for each image based on a continuous number from 0 to 1 corresponding to the probability of that diagnosis and the gastric location of the images. A diagnosis of low risk corresponded to >50% of the images in the antrum and lesser curvature of the gastric body judged as low risk, and a diagnosis of high risk corresponded to >90% of the images in the gastric body and fornix judged as high risk. All other cases were diagnosed as moderate risk.

The same validation dataset was diagnosed as low risk (no atrophy), moderate risk (closed‐type atrophy), or high risk (open‐type atrophy) by three board‐certified specialists at the Japan Gastroenterological Endoscopy Society. The consensus diagnoses of the three endoscopists were made by a majority and were compared with those of the AI system.

### *Statistical analysis*

Quantitative data are shown as median (range). Differences were analyzed using the χ^2^ test, and *P* < 0.05 was considered significant. These analyses were performed on a personal computer using StatView version 5.0 (SAS Institute, Cary, NC, USA). The Cochran–Armitage trend test was performed to assess the trend in the prevalence of GC in each risk group. Interobserver variation in the diagnosis of the risk of GC by three endoscopists was assessed using kappa statistics. A kappa value of >0.8 indicated almost perfect agreement, 0.8–0.6 indicated substantial agreement, 0.6–0.4 indicated moderate agreement, 0.4–0.2 indicated fair agreement, and <0.2 indicated slight agreement. A kappa value of 0.0 indicated agreement equal to chance, and a value of <0.0 suggested disagreement. These analyses were performed using EZR version 1.40 (Saitama Medical Center, Jichi Medical University, Japan).^23^

### *Ethics*

This study was approved by the Institutional Review Board of Osaka International Cancer Institute (no. 2017–1710059178).

## Results

### *Patient characteristics*

The patient characteristics are shown in Table 2. Serum antibody for *H. pylori* was negative in 172 patients, 111 of whom were considered to be *H. pylori*‐uninfected because no sign of gastric atrophy was present.

**Table 2:** Clinical characteristics of the patients in the validation dataset

Clinical characteristics	*n* = 454
Age (years)	67 (33–90)
Gender, *n* (%)	
Male	289 (64)
Female	165 (36)
Body mass index	23.2 (14.1–35.7)
Smoking, *n* (%)	
Never smoker	169 (37)
Past smoker	137 (30)
Current smoker	74 (16)
Unknown	74 (16)
Alcohol drinking, *n* (%)	
Never drinker	120 (26)
Past drinker	27 (6)
Habitual drinker	161 (35)
Social drinker	73 (16)
Unknown	73 (16)
Serum antibody for *Helicobacter pylori*, *n* (%)	
Negative	172 (38)
Positive	282 (62)

### *Performance of AI system for stratifying GC risk*

A total of 12,824 images from 454 patients were included in the analysis. The time required for diagnosing all the images was 345 seconds. The AI system diagnosed 46, 250, and 158 individuals as low‐, moderate‐, and high risk, respectively. No patients fulfilled the criteria for both the low‐ and high‐risk groups. The prevalence of GC in the low‐, moderate‐, and high‐risk groups was 2.2% (1/46), 8.8% (22/250), and 16.4% (26/158), respectively (Table 3). The risk of GC was significantly increased in the moderate‐ and high‐risk groups (*P* = 0.0017).

**Table 3:** Prevalence of gastric cancer in each group diagnosed by AI or board‐certified specialists

Risk	High	Moderate	Low	*P* value
AI	16.4% (26/158)	8.8% (22/250)	2.2% (1/46)	0.00173
Board‐certified specialists	22% (38/173)	6.7% (10/149)	0.76% (1/132)	<0.001

AI, artificial intelligence.

Three board‐certified specialists diagnosed 0.76% (1/132), 6.71% (10/149), and 21.96% (38/173) patients as low‐, moderate‐, and high‐risk, respectively, by a majority. The risk of GC was significantly increased in the moderate‐ and high‐risk groups (*P* < 0.001). Interobserver agreement among the three board‐certified specialists in differentiating the risk was not satisfactory (kappa value of 0.27, indicating fair agreement). Complete agreement regarding the risk of GC was achieved in only 30.2% (137/454) of cases.

### *Characteristics of GCs*

The characteristics of the GCs are shown in Table 4. More than 90% of GCs were early and intestinal‐type cancers. One cancer developed in the low‐risk group in a patient negative for *H. pylori* infection with no atrophy in the stomach, who was therefore considered to be *H. pylori*‐uninfected. This GC was type IIc and 8 mm in diameter, and it was located in the lesser curvature of the antrum. The GC was successfully removed by endoscopic resection, and histologic examination showed intestinal‐type mucosal cancer.

**Table 4:** Clinical characteristics of 49 gastric cancers in the validation dataset

Clinical characteristics	*n* = 49
Location, *n* (%)	
Upper	3 (6)
Middle	28 (57)
Lower	18 (37)
Histopathology type, *n* (%)	
Intestinal type	45 (92)
Diffuse type	4 (8)
Tumor depth, *n* (%)	
Early cancer	46 (94)
Advanced cancer	3 (6)

## Discussion

Premalignant changes in the gastric mucosa are well‐known risk factors for the development of GC^24^ and are included in a widely accepted model leading to intestinal‐type gastric carcinoma. In this multistep model of gastric carcinogenesis, *H. pylori* causes chronic inflammation of the gastric mucosa, which slowly progresses through the premalignant stages of AG, IM, and dysplasia to eventual gastric adenocarcinoma.^5^, ^24^ Other factors, such as xanthelasma,^25^ nodular gastritis,^26^ and enlarged fold gastritis,^27^ have also been reported as risk factors for GC. Comprehensive assessment of these factors may allow accurate stratification of the GC risk.^28^ However, the evaluation of multiple endoscopic images of the stomach may be complicated and are subject to interobserver variability, and such endoscopic assessment is not common practice in areas with a low incidence of GC.

Interobserver variability is a major limitation in the diagnosis of GC by endoscopists. In the present study, three board‐certified specialists from the Japan Gastroenterological Endoscopy Society succeeded in differentiating the risk of GC. However, the interobserver agreement among these three endoscopists was not satisfactory (kappa value of 0.27, indicating fair agreement). Discordance of the risk was confirmed in 69.8% of cases; this may cause confusion in clinical practice. Stratification of GC risk by endoscopists has some problems; therefore, we aimed to develop a system with which to stratify the risk. The current AI system allows objective assessment of the GC risk during endoscopic examination by eliminating the interobserver and intraobserver variability. Based on the risk assessment, the most appropriate intensity of endoscopic observation can be provided (i.e., meticulous observation for high‐risk patients and simplified observation for low‐risk patients), resulting in more effective endoscopic examination. In addition, this risk stratification may guide the surveillance interval. No consensus on the optimal interval of surveillance for GC has yet been established, although a 1–3‐year interval is proposed for patients with gastritis in countries with a high prevalence of GC. The current system could indicate a shorter interval for high‐risk patients and a longer interval for moderate‐ or low‐risk patients.^29^, ^30^, ^31^

In this study, we educated the AI system regarding low‐risk patients using a population with no history of *H. pylori* infection or atrophy. The reported prevalence of GC in patients with no gastric atrophy is 0.05%, while that in patients with gastric atrophy is 1.7%.^32^ Although GC may develop in patients without *H. pylori* infection, this population constituted a large group with minimal risk of GC and was thus considered to be the best material for educating the AI. However, the accuracy of *H. pylori*‐negative results is limited because serum antibody testing reportedly has relatively low sensitivity.^33^ Patients with GC and patients with known risk factors (i.e., spread of GA or IM, originally identified as characteristic findings of the gastric mucosa in patients with GC) could both have been used to educate the system about high‐risk patients. We believed that the best education could be achieved by entrusting the AI system to extract risk features from a large number of mucosal images of GC; therefore, we used images from patients with GC to educate the system regarding high‐risk patients.

Most images used to educate and validate the AI system in the current study were obtained by white‐light imaging. However, narrow‐band imaging (NBI) has recently been reported to be useful in the diagnosis of IM and dysplasia.^34^ NBI or other image‐enhanced endoscopy methods may thus improve the performance of AI for stratifying the risk of GC. We should therefore consider accumulating NBI images of the gastric mucosa to further educate the AI system. This study included endoscopic images obtained by Olympus and Fujifilm systems. The inclusion of two endoscopic systems may impair the accuracy of the AI system. However, considering the generalizability of the AI system, we included two systems and showed acceptable accuracy for stratifying the GC risk.

In this study, the existence of atrophy was determined by endoscopic diagnosis. This is one of the main limitations of our study and may cause concern regarding the accuracy of the classification of risk groups for the training dataset. Confirming the histology of the gastric mucosa may provide more accurate information regarding the mucosal status and raise the accuracy of classification. However, our discrimination between low risk and moderate risk may be reliable because it was made using the information regarding the *H. pylori* infection status in most patients.

Most images of the gastric mucosa, including poor‐quality images, were used in the validation test data. Selecting high‐quality images could have improved the accuracy of the stratification but may have caused bias by selecting characteristic images. We therefore avoided such selection bias by including all images. However, we selected the gastric site for assessment of the GC risk based on the assumption that premalignant changes in the stomach could spread from one part to another. To differentiate the moderate‐ and low‐risk groups, we restricted the images to the antrum and lesser curvature of the gastric body because the initial change of *H. pylori* infection appears in these regions of the gastric body.^35^, ^36^ To differentiate the moderate‐ and high‐risk groups, we restricted the images to the gastric body and fornix because morphological changes in these regions may be more closely related to the risk of GC than those in the antrum.^37^, ^38^ Cut‐off lines to differentiate the low‐ to moderate‐risk groups and the high‐ to moderate‐risk groups were determined to ensure an adequate number of patients in the high‐ and low‐risk groups.

This study had several limitations. First, the cross‐sectional design made it difficult to speculate on the chronological development of GC. Second, the validation data were obtained from patients visiting the cancer hospital with a high prevalence of GC. Further confirmation in the general population is desired, although this would require a large number of people (>10 000). Third, data regarding eradication therapy for *H. pylori* were not shown. Considering the increasing number of patients who undergo *H. pylori* eradication, further studies are needed to develop an AI system specialized for this population.

In conclusion, the current AI system diagnosed GC in 2.2, 8.8, and 16.4% of patients in the low‐, moderate‐, and high‐risk groups, respectively (*P* = 0.007). These results suggest that, despite some limitations, this AI system may be an effective tool for stratifying GC risk.
